# Risk Factors of Noncompliance to Preventive Mass Drug Administration for Eliminating Lymphatic Filariasis: A Case-Control Study in Jawi District, Northwest Ethiopia

**DOI:** 10.1155/2022/4792280

**Published:** 2022-09-21

**Authors:** Fetene Mihretu, Gebeyehu Tsega, Melesse Belayneh, Mesafint Molla Adane

**Affiliations:** ^1^Carter Center Ethiopia, Bahir Dar Regional Project Office, Bahir Dar, Ethiopia; ^2^Department of Health Systems Management and Health Economics, School of Public Health, College of Medicine and Health Sciences, Bahir Dar University, Bahir Dar, Ethiopia; ^3^Department of Environmental Health, School of Public Health, College of Medicine and Health Sciences, Bahir Dar University, Bahir Dar, Ethiopia

## Abstract

**Background:**

High compliance is crucial for the success of a mass drug administration program to achieve lymphatic filariasis elimination. However, the presence of persistently noncompliant individuals might delay the elimination target. Besides, although context-based research is essential to designing effective strategies, only a few studies have focused on identifying factors that play a role in noncompliance with mass drug administration in Africa. Therefore, this study was conducted to identify the factors associated with noncompliance to prevent mass drug administration using ivermectin-with-albendazole for the elimination of lymphatic filariasis in Northwest Ethiopia.

**Methods:**

A case-control study was conducted in Jawi District, Northwest Ethiopia. All individuals who are permanently living in the study area and registered on the annual chemotherapy registration book since 2015 were included in this study. A two-proportion formula was used to estimate the required sample size and 348 cases and 348 controls were selected by identification number on the village chemotherapy registration book using a systematic sampling technique. Data were collected by face-to-face interviews using a structured questionnaire developed through an intensive literature review. Then, data were entered and cleaned by using the EPI DATA software, and analyses were conducted using SPSS version 26. Finally, a logistic regression analysis technique was applied to identify the risk factors using adjusted odds ratio as measures of effect.

**Results:**

A total of 690 (99.1%) participants, 345 cases and 345 controls, were included in the study. Younger age (AOR = 1.60; 95%CI: 1.10, 2.33), female sex (AOR = 1.56; 95%CI: 1.24, 3.93), thought of not being susceptible to the disease (AOR = 2.36, 95%CI: 1.80, 4.32), lack of disease knowledge (AOR = 1.88; 95% CI: 1.38, 3.81), fear of drug side effect (AOR = 2.45; 95% CI:1.23, 4.86), and not participating in community drug distributors selection (AOR = 2.58; 95% CI: 1.70, 3.91) were found to be the risk factors significantly associated with noncompliance.

**Conclusion:**

Noncompliance with lymphatic filariasis mass drug administration therapy was associated with specific demographic, individual, program, and drug delivery characteristics. This finding has important implications for program effectiveness and would be used to accelerate the elimination of lymphatic filariasis in the study area and other endemic settings.

## 1. Introduction

Lymphatic filariasis (LF) infection, commonly known as elephantiasis, is caused by the transmission of the nematode parasites [1, 2] to humans through mosquito bites [3, 4]. It is one of the most debilitating diseases in the world [5], including in Ethiopia [6], that can result in death and disability [7] with high loss of productivity, stigma, and treatment cost [4, 8]. LF is a chronic disease with devastating physical, mental, and socio-economic impacts on the affected individuals [9] and is one of the primary causes of long-term disability [10] with severe effects on the quality of life among Ethiopian families [11]. It is estimated to cause a disease burden of 1.74 million disability-adjusted life years (DALYs) [12], with 51 million people infected worldwide as of 2018 [13].

In Ethiopia, about 35 million people live in endemic areas with a nationwide LF prevalence of 6.2% [14], ranging from 2.8% to 7.4% in different regions of the country [15]. The contemporary strategies to eliminate LF mostly rely on mass drug administration (MDA) which involves giving ivermectin-with-albendazole [16, 17] to the entire population at risk [18]. In recognition of its elimination potential, the Global Program to Eliminate Lymphatic Filariasis (GPELF) was launched by the World Health Organization (WHO) to achieve global elimination by 2030 as a new target year for the elimination of LF as a public health problem [19]. This elimination strategy promoted by GPELF comprises an annual MDA to alleviate the suffering of affected individuals through interrupting transmission, managing morbidity, and preventing disability [4]. Since the commencement of the elimination strategy in 2000, more than 8.6 billion cumulative treatments have been delivered to limit the spread of LF [13]. Nevertheless, the MDA program should cover at least 80% of the individuals at risk to attain the elimination of LF [16], and the elimination objective can be achieved only if issues of drug distribution and implementation challenges are addressed [18].

In Ethiopia, preventive MDA for LF was started in 2009 by community drug distributors (CDDs) [20], and more than 10 rounds of treatments have been administered so far [21]. However, many districts did not achieve the elimination target of 2020 [21], with millions of Ethiopians living in endemic areas [22]. Also, despite the ongoing MDA intervention over a long time, there have been reports of persistent LF transmission in Africa [23, 24] and Ethiopia [21, 22, 25]. Previous mapping studies conducted in different districts of Ethiopia reported a prevalence of 3.7% with high geographical clustering ranging from 0% to more than 50%, and the treatment coverage was reported to be about 81% in 2019 [26]. In particular, the Jawi district remains a hyperendemic area in Ethiopia that failed to meet the WHO critical threshold (Ag <2% and/or MF <1%) for disease elimination after several rounds [21].

Although high coverage and compliance are crucial for the success of an MDA program to achieve elimination [27], the presence of persistently noncompliant individuals (i.e., individuals who commonly missed MDA treatment rounds [28]) might delay the elimination of LF [29]. Also, the effectiveness of this intervention in Africa [30] and Ethiopia [31] is often hampered by several factors. Hence, the high nationwide prevalence [14, 15], the failure to eliminate [21, 25, 32], and the persistent transmission of LF in Ethiopia after a decade of MDA [21, 22, 25], might be linked to the high MDA noncompliance [27]. Furthermore, although context-based research finding is essential to designing effective preventive MDA program strategies [30, 33], only a few studies have focused on identifying factors that play a role in noncompliance to prevent MDA in Africa [30, 31].

Therefore, the current study was conducted to identify the factors associated with noncompliance with preventive MDA of ivermectin-with-albendazole for the elimination of lymphatic filariasis in Northwest Ethiopia. It is hoped that the study will provide a piece of scientific evidence for policymakers, donors, program planners, and implementers about factors associated with noncompliance which can be used to strengthen the MDA program and speed up the elimination of the disease in Ethiopia and other similar settings.

## 2. Methods and Materials

### 2.1. Study Design, Area, and Population

A case-control study was conducted in March 2021. The study area, Jawi district, is located 400 km away from Addis Ababa in Northwest Ethiopia and 150 Km from Bahir Dar city. A total of 146378 (with a male to female ratio of 0.55 to 0.45) population lives in the district within 32528 households. The district has one hospital, six health centers, and 27 health posts. Despite the continuing MDA chemotherapy of ivermectin-with-albendazole for the elimination of LF, the district is still an endemic area for LF with a high prevalence [21].

### 2.2. Inclusion Criteria

All individuals who are permanently living in the study area and have been registered in the annual preventive MDA chemotherapy registration book since 2015 were included in this study. Only individuals with no treatment record in any round due to emigration and individuals who came to the district after the induction of the first preventive MDA chemotherapy were excluded from the study.

### 2.3. Variables Data Sources and Methods of Assessment

This study characterizes cases or noncompliants to LF preventive chemotherapy as individuals who missed at least one dose of the annual preventive MDA chemotherapy of ivermectin-with-albendazole for the elimination of LF since 2015 [34]. Whereas, compliants (controls) were characterized as individuals who took all five rounds of the preventive MDA chemotherapy within the parallel period. The potential predictor variables were broadly categorized into demographic, individual, program, and drug delivery characteristics. The specific predictor variables were assessed through face-to-face interviews with cases and controls by data collectors using questionnaires.

### 2.4. Sample Size Determination

A two-proportion formula (EPI info 7, SATCALC) was used to estimate the sample size required for the study with statistical assumptions of 95% confidence level, a power of 80%, a 1 : 1 ratio of cases and controls, a design effect of 2, a 10% nonresponse rate in both groups, and an OR of 2 to be detected by taking a risk factor of participation in the selection of community drug distributors (CDDs) from an ivermectin treatment adherence study conducted in South Ethiopia with a prevalence of 71.1% and 56% for cases and controls, respectively [34]. Thus, the estimated total sample size was 696 (348 cases and 348 controls).

### 2.5. Sampling Procedure

A multistage random sampling procedure was employed. In the first stage, about 20% of sample villages were selected randomly by lottery method in the district. In the second stage, all eligible cases and controls were listed separately on two different sampling frames from each village's annual preventive MDA chemotherapy registration book. The estimated total sample size was allocated to each village using a probability proportional to size. Then, 348 cases and 348 controls were selected by identification number on the village preventive MDA chemotherapy registration book using a systematic sampling technique.

### 2.6. Data Collection Instruments

Data were collected through face-to-face interviews using a structured questionnaire developed through an intensive literature review. The questionnaire included questions on demographic characteristics (such as age, gender, religion, education, marital status, occupation, and wealth index), individual characteristics such as area of residence, duration of stay in the endemic area, perceived health risk of LF, knowledge of LF, and utilization of long-lasting insecticide-treated net (LLITN), as well as program and drug delivery characteristics (such as participation in the community drug distributor selection, preferred mode of drug delivery, preferred drug dispenser, fear of drug side effects, and perceived capacity of CDDs to distribute drugs).

### 2.7. Data Management and Analysis

Data were entered and cleaned by using the EPI DATA software and analyses were conducted using SPSS Version 26. Each variable was analyzed with the noncompliance of LF treatment in bivariate analysis and those variables with a *P* value <0.2 in bivariate analysis were entered into multivariate stepwise backward logistic regression analysis. Then, the model was tested by the Hosmer and Lemeshow test and it was found to be a good fit model (*P*=0.52). Finally, a logistic regression analysis method was applied to identify the risk factors, using adjusted odds ratios as measures of effect.

### 2.8. Data Quality Assurance

To keep the quality of the data, a standard structured questionnaire was developed in English and translated into Amharic and then back to English to maintain its consistency for actual data collection purposes. One-day training for data collectors and supervisors was conducted by the principal investigator on how to interview and fill out the questionnaire. Data were collected by experienced data collectors who have bachelor's degrees and above in statistics and health. Two individuals with a Master's degree in Public Health background were assigned as supervisors after they had received the necessary training from the principal investigator. Before the actual data collection, the data collection questionnaire was pretested in the nearby district which was not part of the present study area, and the feedback obtained from the pretest was used to correct some unclear ideas and statements. Data coding, data entry, and cleaning were performed to maintain its quality.

## 3. Results

### 3.1. Demographic Characteristics of Participants

A total of 690 participants, 345 cases, and 345 controls, were interviewed, making the response rate 99.1% from the initial selection. The mean age of the respondents was 33.25 years (±SD, 13.39). About 57.7% of respondents were female with 223 (64.6%) cases and 175 (50.7%) controls (Table 1).

### 3.2. Individual, Program, and Drug Delivery Characteristics

Among most of the participants, 81.7% cases and 88.7% controls, have perceived LF as a health problem, and nearly half of the respondents166 (48.1%) cases and 214 (62%) controls had knowledge about the symptoms, transmission, and prevention mechanisms of LF (Table 2).

The majority of respondents 259 (75.1%) cases and 270 (78.3%) controls, have LLITN in their homes and most of them use it. Among the noncompliance individuals for treatment, 4 (1.2%), 17 (4.9%), 44 (12.8%), 134 (38.8%), and 146 (42.3%) of them missed five, four, three, two, and one round of treatments, respectively, out of 5 MDA rounds since 2015. Nearly half of the respondents would like to take drugs at their home, and the majority of the respondents 283 (82) cases and 205 (74.2%) cases had not participated in CDD selection (Table 2).

### 3.3. Factors Associated with Noncompliance to Preventive MDA for the Elimination of Lymphatic Filariasis

An adjusted logistic regression model was developed to identify the factors of noncompliance and prevent MDA. As a result, younger age (AOR = 1.60; 95%CI: 1.10, 2.33), female sex (AOR = 1.56; 95%CI: 1.24, 3.93), thought of not being susceptible to LF (AOR = 2.36, 95%CI: 1.80, 4.32), lack of disease knowledge (AOR = 1.88; 95% CI: 1.38, 3.81), not using long-lasting insecticide-treated nets (AOR = 2.13; 95% CI: 1.18, 3.83), fear of drug side effects (AOR = 2.45; 95% CI:1.23, 4.86), and not participating in community drug distributors (CDDs) selection (AOR = 2.58; 95%CI:1.70, 3.91) were found to be the factors significantly associated with noncompliance to preventive MDA using ivermectin-with-albendazole for the elimination of lymphatic filariasis in Northwest Ethiopia (Table 3). On the other hand, variables such as duration of stay in the endemic area and preferred mode of drug delivery did not show any significant association with noncompliance (Table 3).

## 4. Discussions

Although improving treatment compliance with MDA programs is the key to reaching the goal of interrupting LF transmission [18], the presence of persistently noncompliant individuals may delay the elimination of LF [29]. This study has identified important factors contributing to preventive MDA noncompliance in the study area. Hence, younger age was found more likely to be noncompliant than older age with an estimated AOR of 1.60 (95%CI: 1.10, 2.33). This finding is similar to the study conducted in Tanzania where increasing age was positively associated with noncompliance to LF preventive MDA [35]. This might be due to the fact that as age increases, the health-seeking behavior of an individual increases, thereby improving the person's compliance with MDA treatments.

In the current study, the risk of noncompliance with preventive MDA was found to be more among female clients than among the associated male clients with an estimated AOR of 1.56 (95% CI: 1.24–3.93). The result is consistent with the study conducted in London and Indonesia [36, 37] but different from the study conducted in Tanzania which reported no major difference between females (54.9%) and males (55.8%) [35]. The possible reasons might be that women are usually busy with their domestic duties and might be prohibited by their husbands from participating in MDA because of their husbands' negative beliefs about the drug [33].

In this study, the thought of not being susceptible to the disease was found to be one of the essential factors significantly associated with noncompliance with preventive MDA with an estimated AOR of 2.36 (95%CI: 1.80, 4.32). Likewise, the thought of not being susceptible to LF was significantly associated with noncompliance with MDA in Ghana [AOR = 2.83, 95%CI: 1.15, 6.98] [38]. Therefore, addressing the issue of noncompliance through health education to increase risk perception may be necessary [31].

Clients' knowledge status of LF disease also showed a significant association with noncompliance with preventive MDA. Respondents who had no adequate knowledge about the symptoms, transmission, and prevention of the disease were found to be more noncompliant (AOR = 1.88; 95% CI: 1.38, 3.81) than those who had better knowledge of the disease which is in agreement with the study conducted in London, Ghana, Sir Lanka, and the Philippines [36, 39–41]. A similar study conducted in the Philippines revealed that disease knowledge was highly associated with MDA acceptance (*P*=0.002). Other pieces of literature also reported that lack of disease knowledge was identified as a factor in preventive MDA noncompliance in Ghana [30, 40, 42].

Fear of drug side effects was another predictor of noncompliance in this study. Hence, those clients who reported fear of drug side effects were more likely to be noncompliant with the MDA treatment (AOR = 2.45, 95%CI: 1.23–4.86) than those who did not report fear of drug side effects. Likewise, various studies conducted in different countries showed that the experience of side effects was a predictor variable for noncompliance with MDA treatment. A study conducted in, Ghana, Brazil, India, and Kenya showed that noncompliance was mostly associated with fear of side effects during MDA [30, 40, 43, 44].

As to the participation status in the CDD selection, clients who did not participate in CDD selection were more likely to be noncompliant with MDA (AOR = 2.58; 95% CI: 1.70, 3.91) than those who had participated in CDD selection. A study conducted in Ghana showed that community involvement in the precampaign activities had improved treatment coverage and drug compliance [30]. Similar studies conducted in Ethiopia showed that MDA compliance was influenced by the participation of community members in selecting drug distributors. Those who were involved in the selection of CDDs were more likely to comply with ivermectin treatment as compared to those who did not [34]. The possible explanation might be that participation in the precampaign activities provides an opportunity for improving awareness, building trust, and ownership, leading to improved treatment compliance. Thus, stakeholders should conduct a customized prior community mobilization campaign to increase community participation during MDA drug distributors' selection thereby reducing MDA noncompliance.

### 4.1. Strengths and Limitations of the Study

This study has several strengths such as being a community-based study and training of data collectors with practical exercises among others. However, since the selection of cases and controls entirely depends on the CDTI registration book, this study could be subjected to selection bias. Also, though we made considerable efforts to decrease the possibility of bias in this study, recall biases could be another potential limitation for a survey of this kind.

## 5. Conclusions

Noncompliance with LF preventive MDA therapy was associated with specific demographic, individual recipient, program, and drug delivery characteristics. Age, gender, risk perception, disease knowledge, LLITN utilization, fear of drug side effects, and participation during CDD selection were found to be the major factors significantly associated with noncompliance with preventive MDA using ivermectin-with-albendazole for the elimination of LF. This finding has important implications for the MDA program's effectiveness and can be used to accelerate the elimination of lymphatic filariasis in the study area and other endemic settings. In particular, stakeholders should implement integrated and tailored interventions to improve MDA compliance through raising awareness and increasing community participation during MDA drug distributors' selection. From a future research point of view, conducting further in-depth interviews using a qualitative research method is essential to better understanding the reasons for noncompliance despite having good disease awareness.

## Figures and Tables

**Table 1:** Distribution of respondents by demographic characteristics, Jawi District, Northwest Ethiopia, June 2021 (*n* = 690).

Variables		Cases *n* = 345	Control *n* = 345
Age	<30	206 (59.7)	162 (47)
	≥30	139 (40.3)	183 (53)

Gender	Male	122 (35.4)	170 (49.3)
	Female	223 (64.6)	175 (50.7)

Religion	Orthodox	323 (93.6)	325 (94.2)
	Muslim	22 (6.4)	20 (5.8)

Completed educational level	Do not read and write	193 (55.9)	199 (57.7)
	Read and write only	62 (18)	53 (15.4)
	Primary school (grades 1–8)	45 (13)	45 (13)
	Secondary and above (≥grade 9)	45 (13)	48 (13.9)

Marital status	Married	237 (68.7)	243 (70.4)
	Unmarried	108 (31.3)	102 (29.6)

Occupation	Farmers	215 (62.3)	215 (62.3)
	Employed	49 (48.5)	52 (51.5)
	Unemployed	39 (56.5)	30 (43.5)
	Student	42 (46.7)	48 (53.3)

Gender of household head	Male	293 (84.9)	297 (86.1)
	Female	52 (5.1)	48 (3.9)

Wealth index	Poor	144 (41.7)	138 (40)
	Medium	102 (29.6)	87 (25.2
	Rich	99 (28.7)	120 (34.8)

**Table 2:** Distribution of participants by personal and health provider characteristics, Jawi District, Northwest Ethiopia, June 2021 (*n* = 690).

Variables		Cases *n* = 345	Control *n* = 345
Area of residence	Rural	245 (71)	239 (69.3)
	Urban	100 (29)	106 (30.7)

Duration of stay in endemic the area	<16 years	206 (59.7)	197 (57.1)
	≥16 years	139 (40.3)	148 (42.9)

Perceived LF as a health risk	No	63 (18.3)	39 (11.3)
	Yes	282 (81.7)	306 (88.7)

Overall knowledge of LF (symptoms, transmission, and prevention mechanisms)	Knowledgeable	166 (48.1)	214 (62)
	Not knowledgeable	179 (51.9)	131 (38)

Having LLITNs	No	86 (24.9)	75 (21.7)
	Yes	259 (75.1)	270 (78.3)

Use of LLITNs (*n* = 529; 258 cases and 271 controls)	No	42 (16.2)	22 (8.1)
	Yes	217 (84.8)	248 (91.9)

Getting adequate information on the CDTIA program	No	126 (36.5)	119 (34.5)
	Yes	219 (63.5)	226 (65.5)

Using radio as the main source of information on CDTIA	No	338 (98)	334 (96.8)
	Yes	7 (2)	11 (3.2)

Using television as the main source of information on CDTIA	No	343 (99.4)	338 (98)
	Yes	2 (0.6)	7 (2)

Using CDDs as the main source of information on CDTIA	No	185 (53.6)	175 (50.7)
	Yes	160 (46.4)	170 (49.3

Using HEWs as the main source of information on CDTIA	No	243 (70.4)	250 (72.5)
	Yes	102 (29.6)	95 (27.5)

Preference to take treatments	CDDs	153 (44.3)	187 (54.2)
	HEWs	155 (44.9)	125 (36.2)
	HWs	37 (10.7)	33 (9.6)

If CDDs (*n* = 340)	Female	88 (57.1)	138 (74.2)
	Male	66 (42.9)	48 (25.8)

Experience of drugs side effects	No	285 (82.6)	313 (91)
	Yes	60 (17.4)	31 (9)

The perceived capacity of CDDs to distribute drugs	No	178 (51.6)	142 (41.2)
	Yes	167 (48.4)	203 (58.8)

Participation during CDDs selection	No	283 (82)	205 (59.4)
	Yes	62 (18)	140 (40.6)

Fear of drug side effects	No	315 (91.3)	332 (96.2)
	Yes	30 (8.7)	13 (3.8)

LLITNs, long-lasting insecticide-treated nets; CDDs, community drug distributors, CDTIA, community-directed treatment with ivermectin and albendazole; HEWs, health extension workers.

**Table 3:** Multivariable logistic regression analysis results of the factors for noncompliance to preventive MDA using ivermectin-with-albendazole for the elimination of lymphatic filariasis in Northwest Ethiopia (*n* = 690).

Variables		Cases *n* = 345	Control *n* = 345	COR (95% CI)	AOR (95% CI)
Age	<30	206	162	1.67 (1.24–2.26)	1.60 (1.10–2.33)^*∗*^
	≥30	139	183	1	

Gender	Male	122	170	1	
	Female	223	175	1.78 (1.42–3.06)	1.56 (1.24–3.93)^*∗*^

Religion	Orthodox	323	325	0.90 (0.48–1.69)	1.17 (0.48–2.86)
	Muslim	22	20	1	

Completed educational level	Do not read and write	193	199	1.04 (0.66–1.63)	1.03 (0.54–1.97)
	Read and write only	62	53	1.25 (0.72–2.16)	1.14 (0.55–2.36)
	Primary school (grade 1–8)	45	45	1.08 (0.60–1.91)	0.71 (0.34–1.48)
	Above secondary school (above grade 9)	45	48	1	

Marital status	Married	237	243	1.09 (0.78–1.50)	0.79 (0.50–1.26)
	Unmarried	108	102	1	

Occupation	Farmers	215	215	0.77 (0.45–1.35)	0.57 (0.27–1.21)
	Employed	49	52	1.08 (0.53–2.19)	0.96 (0.41–2.22)
	Unemployed	39	30	0.82 (0.39–1.72)	0.61 (0.25–1.51)
	Student	42	48	1	

Gender of HH head	Female	293	297	0.91 (0.60–1.39)	0.76 (0.52–1.11)
	Male	52	48	1	

Area of residence	Rural	245	239	1.09 (0.78–1.51)	0.73 (0.44–1.21)
	Urban	100	106	1	

Duration of stay in the endemic area	<16 years	206	197	0.90 (0.66–1.22)	0.89 (0.60–1.34)
	≥16 years	139	148	1	

Thought of being susceptible to LF	No	63	39	1.75 (1.14–2.70)	2.36 (1.80–4.32)^*∗*^
	Yes	282	306	1	

Having adequate knowledge of LF	No	179	131	1.76 (1.42–3.77)	1.88 (1.38–3.81)^*∗*^
	Yes	166	214	1	

Having LLITNs	No	86	75	1.19 (0.84–1.70)	1.08 (0.75–1.58)
	Yes	259	270	1	
Use of LLITNs (*n* = 529)	No	42	22	2.18 (1.26–2.77)	2.13 (1.18–3.83)^*∗*^
	Yes	217	248	1	

Preferred mode of drug delivery	Home to home	180	169	1	
	Central	149	157	0.89 (0.65–1.21)	0.78 (0.58–1.38)
	Health facility	16	19	1.01 (0.46–2.21)	0.92 (0.55–2.12)
	Community distributors	2	6	0.31 (0.62–1.57)	0.25 (0.4–1.2)

Preferred drug dispenser	CDDs	153	187	1.49 (1.10–2.01)	1.06 (0.71–1.59)
	Health professional	192	158	1	

Fear of drug side effects	No	315	332	1	
	Yes	30	13	2.43 (1.21–4.80)	2.45 (1.23–4.86)^*∗*^

The perceived capacity of CDDs to distribute drugs	No	178	142	1.52 (1.13–2.06)	1.16 (0.77–1.74)
	Yes	167	203	1	

Participation in CDDs selection	No	283	205	3.12 (2.2–4.42)	2.58 (1.70–3.91)^*∗*^
	Yes	62	140	1	

## Data Availability

The data supporting the current study are available from the corresponding author upon request.

## Abbreviations

ALB: Albendazole
AOR: Adjusted odds ratio
CDD: Community drug distributor
CDTIA: Community-directed treatment with ivermectin and albendazole
CI: Confidence interval
COR: Crude odds ratio
DALYs: Disability-adjusted life years
FMoH: Federal Ministry of Health of Ethiopia
GPELF: Global program to eliminate lymphatic filariasis
HEW: Health extension worker
IVM: Ivermectin
LF: Lymphatic filariasis
LLITN: Long-lasting insecticide-treated net
LMICs: Low- and middle-income countries
MDA: Mass drug administration
NTD: Neglected tropical diseases
SSA: Sub-Saharan Africa
WHO: World Health Organization
WoHO: Woreda health office.
